# Supplementary material for: The Anti-Inflammatory and Skin Barrier Function Recovery Effects of Carica papaya Peel in Mice with Contact Dermatitis
Source: Int J Mol Sci. 2025 Nov 17;26(22):11122. doi: 10.3390/ijms262211122 (PMC12653787; doi:10.3390/ijms262211122)
Supplement: Supplementary file 1 [file ijms-26-11122-s001.zip › Supplementary data S1. Body weight and Spleen body weight ratio.pdf]

Supplementary data S1

Measurement of Body Weight and Spleen/Body Weight Ratio

Body weights of individual mice were measured on day 1 and day 15 using an electronic balance (CAS, Gyeonggi, Korea). Changes in body weight were calculated as a percentage relative to the day 1 measurement. Spleens were excised and weighed on day 15 using microbalance (Sartorius, Göttingen, Germany). The spleen body weight ratios were determined by dividing the spleen mass by the corresponding body weight.

EECP Treatment Had No Significant Impact on Spleen Size

To evaluate the effect of EECP on spleen enlargement, spleen body weight ratios were assessed. The ratios observed in the EECP-treated groups showed no notable differences compared to the CTL group. In contrast, treatment with DEX resulted in a significant reduction in the spleen body weight ratio (Supplementary Data S2, Figure S2).

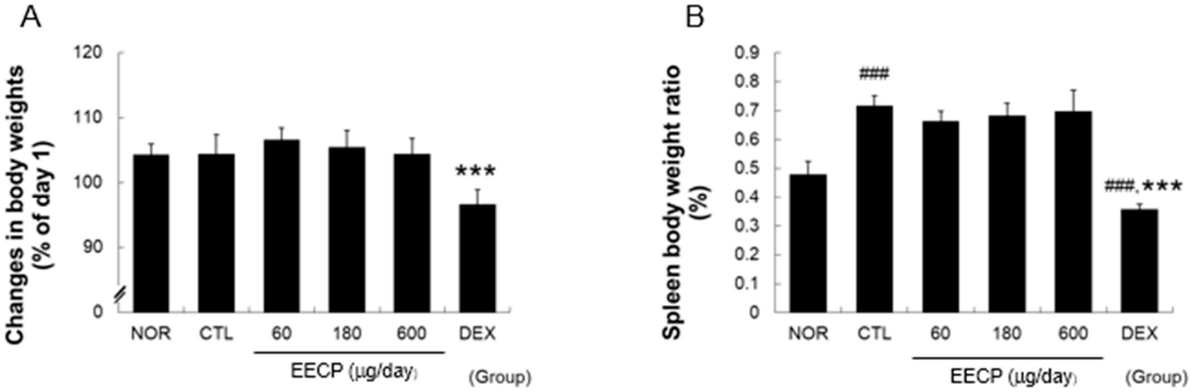

Figure S1. The effects of EECP on DNFB-induced alterations in body weight and spleen body weight ratio. A, changes in body weights; B, spleen body weight ratio. All values are expressed as the mean ± SDs. $^{###}P < 0.001$ vs. NOR; $^{***}P < 0.001$ vs. CTL.
