# Supplementary material for: The Anti-Inflammatory and Skin Barrier Function Recovery Effects of Carica papaya Peel in Mice with Contact Dermatitis
Source: Int J Mol Sci. 2025 Nov 17;26(22):11122. doi: 10.3390/ijms262211122 (PMC12653787; doi:10.3390/ijms262211122)
Supplement: Supplementary file 1 [file ijms-26-11122-s001.zip › Supplementary data S2. Effects on the Normal Skin.pdf]

Supplementary data S2

Effects of Topical Application of *Carica papaya* Peel Extract on the Normal Skin of Mice

1. Introduction

Carica papaya L. is rich in bioactive compounds, including flavonoids and sterols, which have demonstrated anti-inflammatory, anti-arthritic, antimicrobial, and wound-healing activities in preclinical studies. These findings suggest that papaya extracts may offer therapeutic potential for inflammatory conditions. However, clinical evidence and standardized formulations remain limited, highlighting the need for further research to establish their efficacy and safety.

This study was designed to investigate the effects of topical application of *C. papaya* peel on normal skin. The experimental parameters considered included skin lesion severity, skin weight, skin color measurements (erythema and melanin indices), and skin hydration status (water content and water-holding capacity, WHC). In addition, changes in body weight and spleen weight were also examined in balb/c mice.

2. Results

2.1. EECP did not induce any visible symptoms on the skin surface.

Topical application of EECP at a dose of 600 μg/day for 6 days did not cause any noticeable skin surface symptoms, similar to the normal group treated with vehicle only (Figure S2A). In addition, EECP did not have any significant effect on skin thickness (Figure S2B).

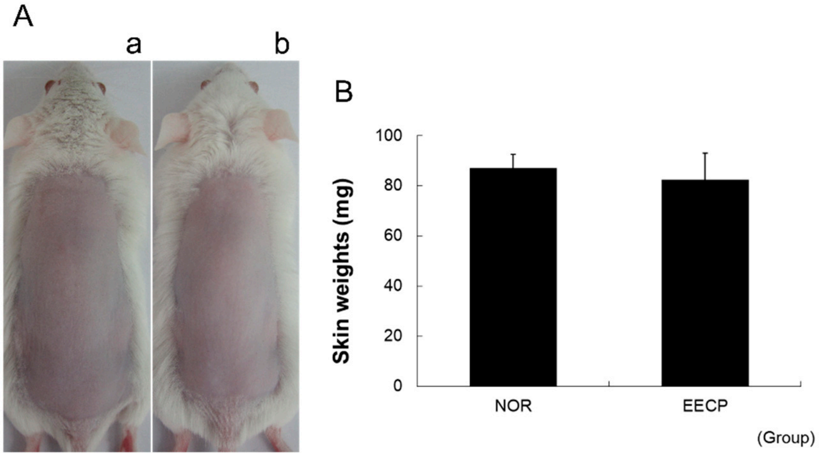

Figure S2. The effect of EECP on skin lesions and skin weights in normal mice. A, non-treated normal mice (NOR); b, 600 μg/day of EECP (A). Skin weights were measured using a microbalance on day 11. EECP, ethanol extract of *C. papaya* peel. Values are expressed as means ± SDs (B).

2.2. EECP did not affect skin color in normal mice.

The erythema and melanin indices in the EECP-treated group for 6 days were similar to those of the normal group treated with vehicle only (Figure S3).

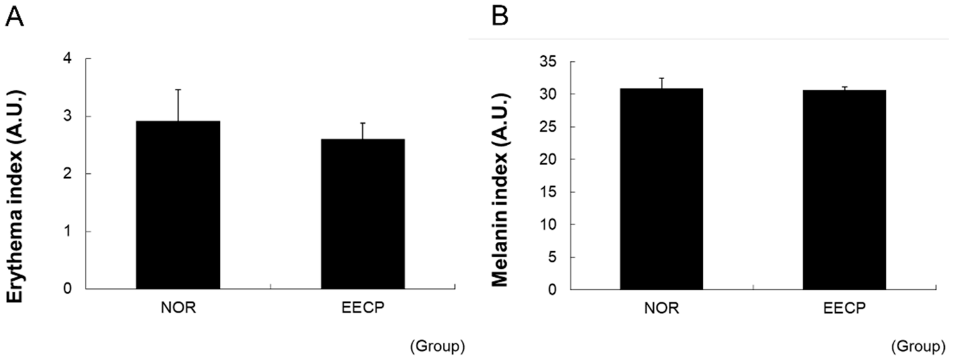

Figure S3. The effects of EECP on erythema and melanin indices. Erythema (A) and melanin indices (B) were quantified using a skin colorimeter. NOR, non-treated normal mice; EECP, ethanol extract of *C. papaya* peel. Values are expressed as means ± SDs.

2.3. EECP increased skin water content and water holding capacity.

Topical application of EECP for 6 days significantly increased skin moisture content and WHC compared with the normal group treated with vehicle only (Figure S4).

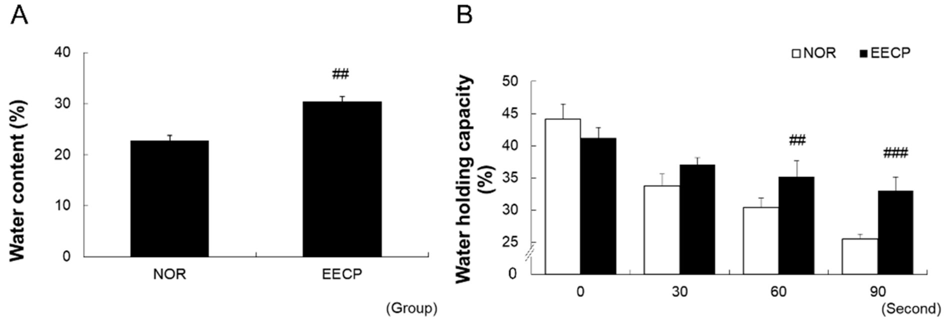

Figure S4. The effects of EECP on skin water content and WHC. (A) Water content; (B) Water holding capacity. NOR, non-treated normal mice; EECP, ethanol extract of *C. papaya* peel. Values are expressed as means ± SDs. ##P < 0.01 vs. NOR and ###P < 0.001 vs. NOR.

2.4. EECP treatment had no significant impact on body weight and spleen size

In the normal mice treated with vehicle only, body weight increased by 10.6% over 11 days, while the EECP-treated group showed a 9.2% increase. No statistically significant differences were observed on any of the measurement days (Figure S5A).

To evaluate the effect of EECP on spleen enlargement, spleen body weight ratios were assessed. The ratios observed in the EECP-treated groups showed no notable differences compared to the NOR group (Figure S5B).

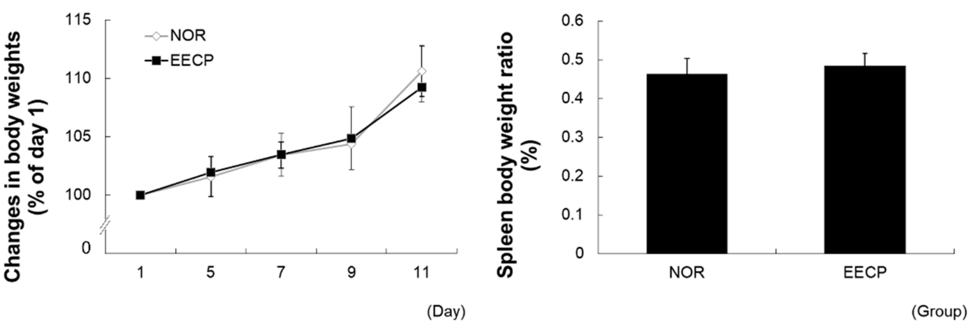

Figure S5. The effects of EECP on changes in body weight and spleen body weight ratio. A, changes in body weights; B, spleen body weight ratio. Values are expressed as means ± SDs.

3. Discussion

The results of this study showed that topical application of EECP at 600 μg/day—the highest concentration used in previous efficacy studies—for 6 days did not induce any notable symptoms on the skin surface and had no significant effect on skin thickness (Figure 1). In addition, it did not affect skin redness or pigmentation levels (Figure 2). Although limited, these findings suggest that EECP does not cause any noticeable skin irritation within the 600 μg/day dose range, at which it exhibits anti-inflammatory and skin barrier–improving effects.

Interestingly, topical application of EECP for 6 days resulted in a significant increase in skin moisture content and WHC compared with the normal mice treated with vehicle only (Figure 3). These results suggest that EECP may enhance skin barrier function even under normal conditions where the barrier is functioning properly.

As shown in Figure 4, EECP did not have any noticeable effects on body weight changes or spleen size in normal mice. Although not directly compared in this study, this observation contrasts with the spleen atrophy commonly induced by corticosteroids. In addition, based on our observations of various behaviors such as feeding activity, fur glossiness, and any abnormal signs around the eyes and nose, EECP did not cause any apparent adverse effects within the limits of our observation.

4. Materials and Methods

4.1. Preparation of the papaya peel extract

Papaya fruit (Green papaya, Dole, Philippines) was purchased through an internet vendor (Coupang, Seoul, Korea). Peel was obtained after washing and removing the flesh, and then dried in a forced convection oven (JSOF-150, JSR, Chungnam, Korea) at 60 °C for 24 hrs. Extractions were performed using a standard laboratory procedure. Briefly, dried papaya peel (100 g) was soaked in 500 mL of 70% ethanol, sonicated for 5 min, and extracted for 24 h. The supernatant was then collected, and the peel was subjected to a second extraction using an additional 500 mL of 70% ethanol for another 24 h after 5 minutes of sonication. The extract was then filtered through Whatman No. 20 filter paper, concentrated using a rotary evaporator (Eyela, Tokyo, Japan), and freeze-dried (Labconco, Kansas City, MO, USA). This process yielded 9.31 g of lyophilized extract (9.31% yield). A sample of this ethanol extract of C. papaya peel (EECP, voucher No. MS2022-1029) was stored at the Division of Pharmacology, School of Korean Medicine, Pusan National University (Supplementary Data S2, Figure S2).

4.2. Animals

Animal experiments were performed using 7-week-old male Balb/c mice obtained from Hana Biotech (Gyeonggi-do, Korea). Animals were maintained in a specific pathogen-free (SPF) environment under a controlled 12-hour light/dark cycle with free access to standard laboratory chow and water. All experimental protocols were reviewed and approved by the Institutional Animal Care and Use Committee (IACUC) of Pusan National University (Approval No. PNU-2022-0208; August 8, 2022), in accordance with institutional and national guidelines.

4.3. Experimental design

Animals were randomly divided into two groups: the vehicle treated-naive group (NOR, n = 5), three EECP treatment group (600 μg/day; n = 6). On day 1, dorsal hair was removed, the mice were left undisturbed for 3 days to allow for the recovery of minor wounds. EECP (10 mg/mL; 60 μL/day) or vehicle (EAOO; ethanol:AOO, 4:1) were applied to shaved backs once daily from day 5 to day 10. All mice were sacrificed on day 11. EECP was dissolved in ethanol and then diluted in AOO (EAOO). The summary of the experimental design is provided in Figure 5.

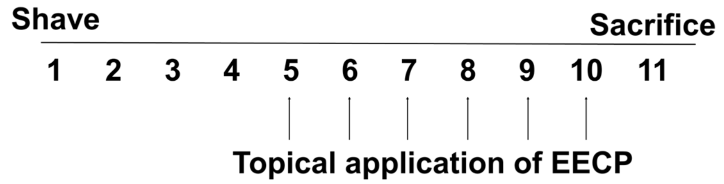

Figure S6. Experimental schedule.

4.4. Skin observation and assessment of skin lesions and weight

Photographs were taken of the shaved dorsal skin of all mice using a digital camera (IXUS 990 IS, Canon, Oita, Japan) on day 11. On the same day, skin samples (5 mm diameter) were collected using a biopsy punch and weighed using a microbalance (Sartorius AG, Germany).

4.5. Assessment of skin color

Skin color was assessed on day 11. Erythema and melanin levels were measured at three different sites per mouse using a skin colorimeter (DSM II, Cortex Technology, Horsens, Denmark), and skin color was calculated by averaging these measurements.

4.6. Skin water content and water-holding capacity

Skin water content and WHC were evaluated using a skin hygrometer (Scalar Corporation, Tokyo, Japan) at 20°C and 20% relative humidity before sacrifice (on day 11). Briefly, skin water contents were assessed at three dorsal locations per mouse. WHC values were determined by measuring skin water content four times at 30-second intervals. The first measurement was taken immediately after removing a wet gauze (1×1 cm, soaked in distilled water) that had been placed on the shaved dorsal skin for 30 seconds.

4.7. Measurement of body weight and spleen/body weight ratio

Body weights of individual mice were measured on days 1, 5, 7, 9 and 11 using an electronic balance (CAS, Gyeonggi, Korea). Changes in body weight were calculated as a percentage relative to the day 1 measurement. Spleens were excised and weighed on day 11 using microbalance (Sartorius, Göttingen, Germany). The spleen body weight ratios were determined by dividing the spleen mass by the corresponding body weight.

4.8. Statistical analysis

Statistical analysis was performed using the *t*-test and the Mann–Whitney U test to assess the significance of intergroup differences. All analyses were performed with GraphPad Prism version 5.01 for Windows (GraphPad Software Inc., La Jolla, CA, USA). Results are presented as mean values ± standard deviations (SDs), and statistical significance was defined as $p < 0.05$.

5. Conclusions

Topical application of EECP at 600 μg/day for 6 days did not cause skin irritation or adverse systemic effects in normal mice, while enhancing skin moisture content and WHC. These findings indicate that EECP is safe within this dose range and may improve skin barrier function even under normal physiological conditions.
