# Supplementary material for: The Anti-Inflammatory and Skin Barrier Function Recovery Effects of Carica papaya Peel in Mice with Contact Dermatitis
Source: Int J Mol Sci. 2025 Nov 17;26(22):11122. doi: 10.3390/ijms262211122 (PMC12653787; doi:10.3390/ijms262211122)
Supplement: Supplementary file 1 [file ijms-26-11122-s001.zip › Supplementary data S3. Components analysis using HPLC.pdf]

Supplementary data S3

Component verification through HPLC

1. Materials and methods

1.1. Chemicals and reagents

HPLC-grade methanol, acetonitrile, and water were purchased from J.T. Baker Inc. (Phillipsburg, USA). Trifluoroacetic acid (TFA) was purchased from Sigma-Aldrich (St Louis, MO, USA). Quercetin, kaempferol and isorhamnetin were purchased from ChemFace (Wuhan, Hubei, China).

1.2. Preparation of standard solution and sample solution

Quercetin, kaempferol and isorhamnetin were accurately weighed and dissolved in the methanol at the concentrations of 1000 μg/mL to make a standard solution. Sample (EECP) was diluted with the HPLC-grade methanol and then filtered through a 0.2 μm syringe filter (BioFACTTM, Korea) prior to HPLC injection.

1.3. Chromatography and mass spectrometry

Qualitative analysis was performed using Agilent 1200 series (Agilent Technologies, Palo Alto, CA, USA) and data was acquired using ChemStation (Agilent Technologies, Palo Alto, CA, USA). A Capcell Pak Mg II C18 column (4.6 mm × 250 mm, 5 μm; Shiseido, Tokyo, Japan) was used for separation of analytes in the column oven at 35 °C. The flow rate was set at 1 mL/min and the injection volume was set at 10 μL. Gradient elution system was applied with the mobile phase consisted of water containing 0.1% trifluoroacetic acid (A) and acetonitrile (B): 10–10% (B) over 0–2 min, 10–80% (B) over 2–25 min, held for 2 min, 80–10% (B) over 27–35 min, and then re–equilibrated to 10% until 40 min. The detection wavelengths were set at UV 340 nm.

2. Results

2.1. Identification of quercetin, kaempferol and isorhamnetin in the sample

The peak of quercetin was detected in the sample (tR: 12.452 min), kaempferol was detected in the sample (tR: 14.230 min) and that of isorhamnetin peak was detected in the sample (tR: 14.551 min) (Figure S7A and S7B), which were identified using the retention time and the UV spectrum of each standard compound.

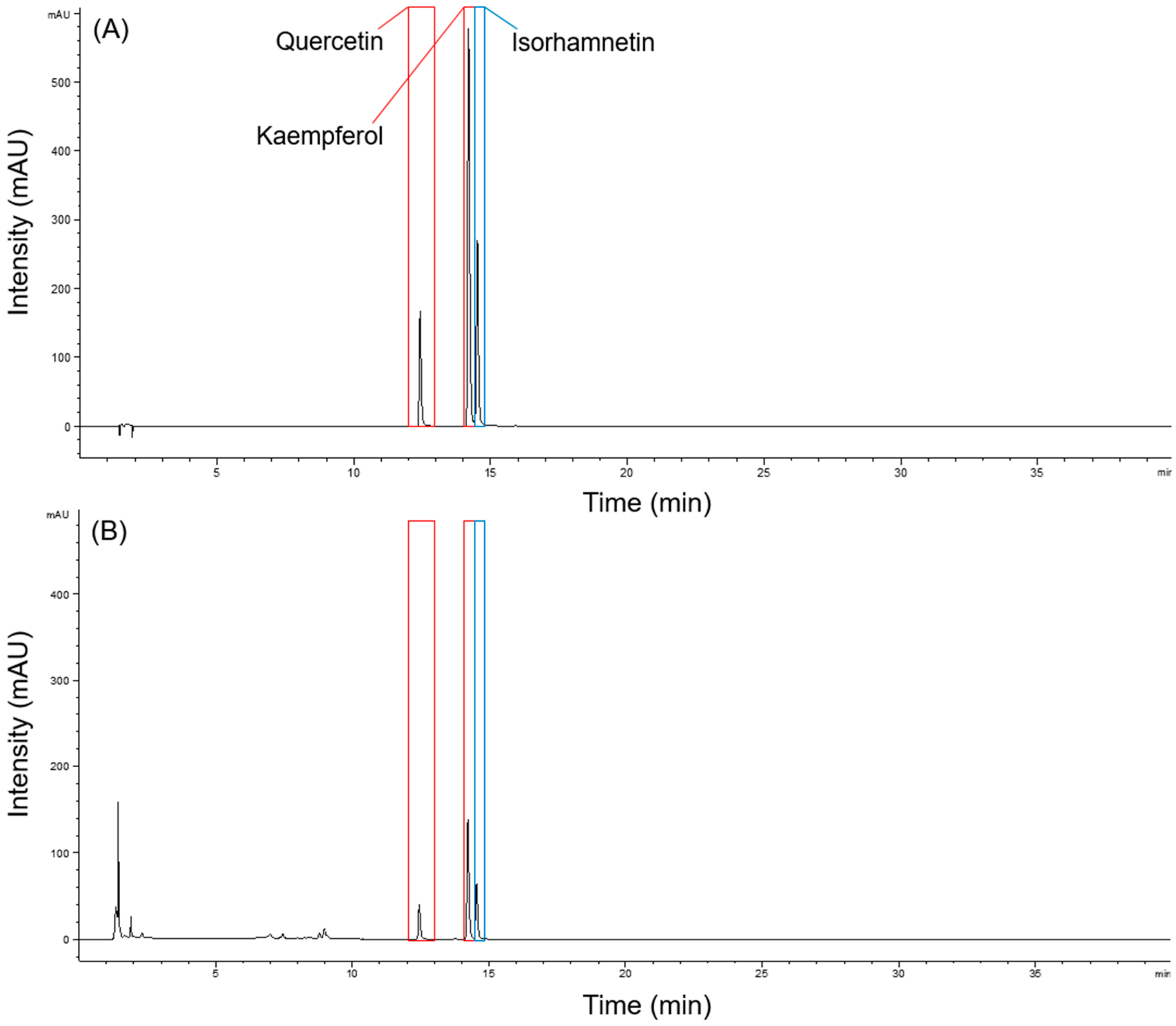

Figure S7. Chromatograms of EECP. Chromatograms of Quercetin, kaempferol and isorhamnetin mixture (A), the sample solution (B) detected at the UV 340 nm.
