# Supplementary material for: The Anti-Inflammatory and Skin Barrier Function Recovery Effects of Carica papaya Peel in Mice with Contact Dermatitis
Source: Int J Mol Sci. 2025 Nov 17;26(22):11122. doi: 10.3390/ijms262211122 (PMC12653787; doi:10.3390/ijms262211122)
Supplement: Supplementary file 1 [file ijms-26-11122-s001.zip › Supplementary data S4. Experimental design.pdf]

Supplementary data S4

Induction of CD and Experimental Design

Animals were randomly divided into six groups: the treatment-naive group (NOR, n = 5), the CD control group (CD CTL, n = 8), three EECP treatment groups (60, 180, and 600 μg/day; n = 8 per group), and the dexamethasone-treated positive control group (DEX, n = 8). All animals, except those in the NOR group, were sensitized by applying 10 μL of DNFB (0.1%, v/v) dissolved in AOO (acetone:olive oil, 4:1) to both ears for three consecutive days. On day 5, dorsal hair was removed, and DNFB (0.1%, v/v; 40 μL/day) was applied to the dorsal skin on days 8, 10, 12, and 14 to induce CD. The NOR group was treated with AOO on the same manner. Topical treatments with EECP (1, 3, or 10 mg/mL; 60 μL/day) or DEX (2.5mg/mL; 60 μL/day) were applied to the shaved back daily from days 9 to 14. EECP and DEX were dissolved in ethanol and then diluted in AOO (EAOO; ethanol:AOO, 4:1). The NOR and CD CTL groups were treated with EAOO on the same manner. On days when both DNFB and either EECP or DEX were administered, DNFB was applied first, followed by EECP or DEX after more than 4 hours. All animals were sacrificed on day 15. The experimental process is summarized in Supplementary Data (Supplementary Data S4, Figure S8).

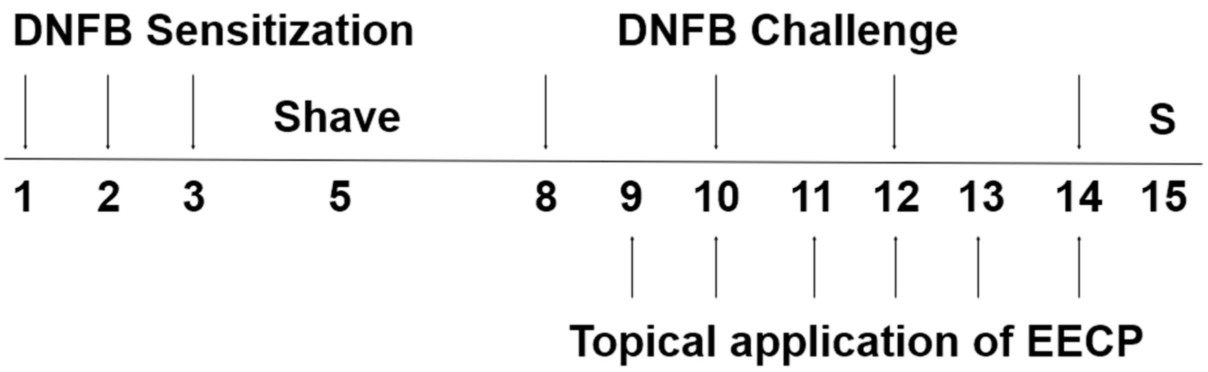

Figure S8. Experimental schedule. S means sacrifice.
