# Supplementary material for: The Anti-Inflammatory and Skin Barrier Function Recovery Effects of Carica papaya Peel in Mice with Contact Dermatitis
Source: Int J Mol Sci. 2025 Nov 17;26(22):11122. doi: 10.3390/ijms262211122 (PMC12653787; doi:10.3390/ijms262211122)
Supplement: Supplementary file 1 [file ijms-26-11122-s001.zip › Supplementary data S5. The primer sets for qPCR.pdf]

Supplementary data S5

Table S1. Target primers used for qPCR

Target gene	Primer sequences (5' to 3' direction)
ICAM-1	Forward: CATCGGGGAATCAGTGACTG<br>Reverse: TGGGCCTGTTGTAGTCTGGG
IL-6	Forward: CAGACAGCCACTCACCTCTT<br>Reverse: CTTTTTCAGCCATCTTTGGA
IL-8	Forward: GTTTTGCCAAGGAGTGCTAA<br>Reverse: CCAGACAGAGCTCTCTTCCA
MCP-1	Forward: CATCCAGAGCTTGAGTGTGA<br>Reverse: GTTAGCCTTGCCTTTGTTCA
GAPDH	Forward: AGGTCGGAGTCAACGGATTT<br>Reverse: ATCTCGCTCCTGGAAGATGG
